# Polymyxins as Novel and Safe Mucosal Adjuvants to Induce Humoral Immune Responses in Mice

**DOI:** 10.1371/journal.pone.0061643

**Published:** 2013-04-11

**Authors:** Naoto Yoshino, Masahiro Endo, Hiroyuki Kanno, Naomi Matsukawa, Reiko Tsutsumi, Ryosuke Takeshita, Shigehiro Sato

**Affiliations:** 1 Division of Infectious Diseases and Immunology, Department of Microbiology, School of Medicine, Iwate Medical University, Iwate, Japan; 2 Division of Leading Pathophysiology, Department of Pathology, School of Medicine, Iwate Medical University, Iwate, Japan; 3 Department of Obstetrics and Gynecology, School of Medicine, Iwate Medical University, Iwate, Japan; Instituto Butantan, Brazil

## Abstract

There is currently an urgent need to develop safe and effective adjuvants for enhancing vaccine-induced antigen-specific immune responses. We demonstrate here that intranasal immunization with clinically used polypeptide antibiotics, polymyxin B (PMB) and colistin (CL), along with ovalbumin (OVA), increases OVA-specific humoral immune responses in a dose-dependently manner at both mucosal and systemic compartments. Enhanced immunity by boosting was found to persist during 8 months of observation. Moreover, mice intranasally immunized with OVA plus various doses of PMB or CL showed neither inflammatory responses in the nasal cavity and olfactory bulbs nor renal damages, compared to those given OVA alone. These data suggest that polymyxins may serve as novel and safe mucosal adjuvants to induce humoral immune responses. The polymyxin adjuvanticity was found to be independent of endotoxins liberated by its bactericidal activity, as indicated by similar enhancing effects of PMB in lipopolysaccharide (LPS)-hyporesponsive and LPS-susceptible mice. However, despite the presence of preexisting anti-PMB antibodies, we observed no reduction in the adjuvant function of polymyxins when they were given intranasally. Furthermore, the titers of OVA-specific Abs in mice intranasally immunized with OVA plus PMB or CL were significantly higher than those in mice administered with polymyxin analogues, such as polymyxin B nonapeptide and colistin methanesulfonate. The levels of released β-hexosaminidase and histamine in mast cell culture supernatants stimulated by PMB or CL were also significantly higher than those stimulated by their analogues. These results suggest that both the hydrophobic carbon chain and hydrophilic cationic cyclic peptide contribute to the mucosal adjuvanticity of PMB and CL.

## Introduction

Since most infectious agents are transmitted via mucosal surfaces, vaccines are generated to induce protective immunity in mucosal tissues, along with external secretions containing specific antibodies (Abs) that would act as the first line of defense at the initial invasion site. The protective immune responses at mucosal surfaces can also be enhanced by coadministration of effective mucosal adjuvants. For instance, enterotoxins, such as cholera toxin (CT) and heat-labile toxin (LT), are powerful mucosal adjuvants that enhance both mucosal and systemic immune responses to coadministered protein antigens (Ags) [1]–[3]. However, safety concerns were aroused when a commercial intranasal vaccine using LT as an adjuvant was withdrawn from the market due to a possible association with side effects, including rhinorrhea, headache, and, most seriously, facial palsy [4]. To develop safer mucosal adjuvants, we focused on existing drugs that have already been tested in humans and are therefore relatively safe.

Interestingly, mast cells have recently come into the limelight as a new player in Ag-specific adaptive immune responses induced by vaccination [5], [6]. These cells interact with various immune cells, such as lymphocytes [7], [8], macrophages [9], dendritic cells [10]–[12], and Langerhans cells [13], and their activation not only initiates the innate immune responses but also elicits the migration of immune cells into draining lymph nodes, thereby inducing the adaptive immune responses [7], [12], [13]. Cationic peptides, on the other hand, are potent substances involved in many aspects of innate immunity [14], [15]. For example, some cationic peptides have been shown to stimulate chemoattraction of monocytes [16], [17] and neutrophils [18], [19], while others promote nonopsonic phagocytosis by macrophages [20]. Based on these findings, mast cell activators and cationic peptides have been considered as a new category of vaccine adjuvants.

Polymyxins isolated from *Bacillus polymyxia* [21] are well-known clinically used antibiotics. Among them, polymyxin B (PMB) and polymyxin E, also called colistin (CL), have been used in clinical practice since 1950s. Polymyxins are highly bactericidal against gram-negative organisms and are used for treating infections caused by multidrug-resistant (MDR) *Pseudomonas aeruginosa*, *Acinetobacter baumannii*, and *Klebsiella pneumoniae* [22], [23]. In addition, polymyxins have long been recognized as mast cell activators, because they were shown to induce not only the degranulation of mast cells *in situ* [24], [25] but also a release of histamine and other mediators from peritoneal cells and isolated mast cells *in vitro* [26], [27].

In the current study, we examined the mucosal adjuvanticity of polymyxins in the induction of coadministered Ag-specific humoral immune responses by intranasal administration, while guarding against toxic side effects with the use of existing drugs.

## Materials and Methods

### Ethics Statement

This study was carried out in strict accordance with the recommendations in the Guidelines for Proper Conduct of Animal Experiments established by the Science Council of Japan, based on the Law for the Humane Treatment and Management of Animals (Law No. 105, 1973) and the Standards Relating to the Care and Management of Experimental Animals (Notice No. 6 of the Prime Minister's Office 1980). The protocol was approved by the Committee on the Ethics of Animal Experiments of Iwate Medical University (Permit No. 22-003 and 23-026). Mice were maintained in the experimental animal facility under pathogen-free conditions for more than 1 week before being used in experiments. All surgery was performed under ketamine anesthesia, and all efforts were made to minimize suffering.

### Candidate Adjuvants and Antigen

**Figure 1:**
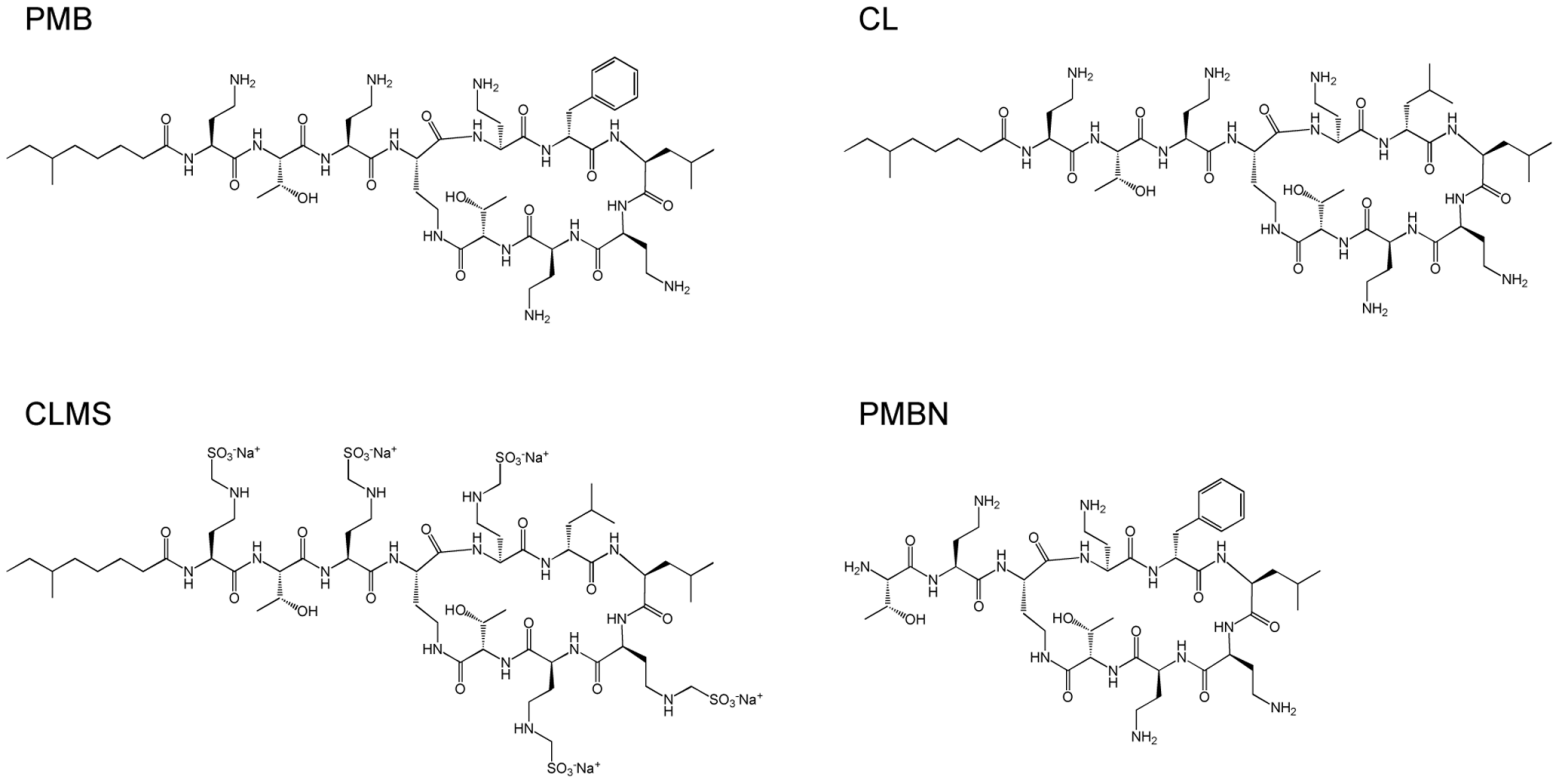
Structures of polymyxins and their analogues. PMB, polymyxin B sulfate; CL, colistin sulfate; CLMS, colistin sodium methanesulfonate; PMBN, polymyxin B nonapeptide hydrochloride.

Ovalbumin (OVA; Sigma-Aldrich, St Louis, MO) was employed as an Ag in all experiments. PMB sulfate, CL sulfate, and CL sodium methanesulfonate (CLMS) obtained from Wako Chemical Industries, Ltd. (Osaka, Japan), as well as PMB nonapeptide hydrochloride (PMBN) from Sigma-Aldrich, were assessed for mucosal adjuvanticity. The structures of polymyxins and their analogues are shown in Fig. 1. All candidate adjuvants and OVA were dissolved in normal saline (<0.25 endotoxin units/mL; Otsuka Pharmaceutical Factory, Inc., Tokushima, Japan).

### Mice and Immunization

Five-week-old C57BL/6N, C3H/HeN, and C3H/HeJ female mice were purchased from the CLEA Japan, Inc. (Tokyo, Japan). Mice were lightly anesthetized with ketamine before being immunized with 5-µL aliquots (2.5 µL/nostril) of normal saline containing 100 µg of OVA and various dosages of candidate adjuvants, while being careful about curarization [28]. All groups of mice were immunized thrice with weekly intervals, the schedule of which was largely the same as that used in previous studies [3], [29]–[31].

### Sample Collection and Preparation of Single-Cell Suspensions

Mucosal secretions (fecal extracts, nasal washes, saliva, and vaginal washes) and blood samples collected using methods described elsewhere [32]–[34] were stored at −80°C until use. Single-cell suspensions were obtained as previously described [34] from the nasal lamina propria (n-LP), nasopharynx-associated lymphoid tissue (NALT), small intestinal lamina propria (i-LP), mesenteric lymph nodes (MLNs), submandibular glands (SMGs), submandibular lymph nodes (SMLNs), spleen, and axillary lymph nodes (ALNs).

### Detection of OVA-Specific Ab and Enumeration of OVA-Specific Ab-Forming Cells

Titers of OVA-specific Abs in plasma and mucosal secretions were determined using an endpoint ELISA. The endpoint titers were the last dilutions that displayed an optical density at 450 nm (OD_450_) of ≥0.1 OD units above the negative controls [30], [32]. For each experiment, vaginal washes were pooled from 4 mice. The numbers of OVA-specific IgA and IgG Ab-forming cells (AFCs) in mouse tissues were determined by an enzyme-linked immunosorbent spot (ELISPOT) assay. OVA-specific AFCs were quantitated with the aid of a stereomicroscope, as described previously [30], [32].

### Detection of PMB-Specific Abs

Titers of PMB-specific IgG and IgM Abs in plasma were determined by an endpoint ELISA. Briefly, serial dilutions of plasma samples were loaded into 96-well plates (Nunc A/S, Roskilde, Denmark) precoated with 1 mg/mL of PMB, followed by blocking with ELISA Ultrablock (AbD Serotec, Oxford, UK). Mouse anti-PMB IgM Ab (clone 45; Thermo Fisher Scientific Inc., Rockford, IL) was used as a positive control. After incubation, horseradish peroxidase-conjugated goat anti-mouse IgG or IgM Abs (Southern Biotechnology Associates, Inc., Birmingham, AL) were added to each well. Colorimetric reactions were developed with the TMB+ Substrate-Chromogen (DAKO, Carpinteria, CA) and terminated by addition of 0.5 M H_2_SO_4_. Plates were then read at 450 nm by a microplate reader (Tecan Group Ltd., Männedorf, Switzerland).

### Determination of IgE Abs and Histopathologic Analysis

We assessed concentrations of total IgE Abs in plasma, as well as inflammatory responses in mouse nasal mucosa, olfactory bulbs, and kidneys. One week after the last immunization, concentrations of IgE Abs in plasma were determined using a sandwich ELISA [35]. Histological sections were prepared and scored for inflammatory grade as described elsewhere [30].

### β-Hexosaminidase and Histamine Assay

MC/9 cells, a murine mast cell clone derived from mouse fetal liver (American Type Culture Collection, Manassas, VA) [36], were maintained in a 10% CO_2_ humid atmosphere at 37°C in Dulbecco's Modified Eagle Medium, supplemented with 10% heat-inactivated fetal calf serum, Rat T-STIM with Con A (BD Biosciences, San Jose, CA), and 0.05 mM 2-mercaptoethanol. For a degranulation assay, MC/9 cells (2×10^5^ cells/200 µL per well) were cultured with or without various dosages of PMB, CL, PMBN, CLMS, and compound 48/80 (a positive control; Sigma-Aldrich) for 30 min in 96-well microplates containing Tyrode's salt solution (Sigma-Aldrich). After centrifugation, supernatants were collected and degranulation was determined by a β-hexosaminidase assay as described previously [37], [38]. Briefly, supernatant samples were incubated at 37°C for 60 min with 1 mM 4-nitrophenyl *N*-acetyl-β-D-glucosaminide (Sigma-Aldrich) in a 0.1 M citric acid buffer (pH 4.5). Reactions were stopped by adding 2 M glycine buffer (pH 10.4), and absorbance at 405 nm was measured by a microplate reader. The release of total intracellular mediators was achieved by cell lysis with 0.2% Triton X-100 in Tyrode's salt solution. The percentages of released β-hexosaminidase were calculated by a formula described elsewhere [38]. Concentrations of histamine in each supernatant sample were determined by a histamine EIA kit (SPI-Bio, Montigny le Bretonneux, France).

### Statistical Analysis

Normally distributed variables were compared using the 2-tailed Student's *t*-test, and results were expressed as mean ± standard deviation (SD). Non-normally distributed variables were compared using the 2-tailed Mann-Whitney U test, and results were expressed as median ± interquartile range (IQR). A *p* value of <0.05 was considered significant.

## Results

### Assessment of OVA-Specific Humoral Immunity in Mucosal and Systemic Sites

**Figure 2:**
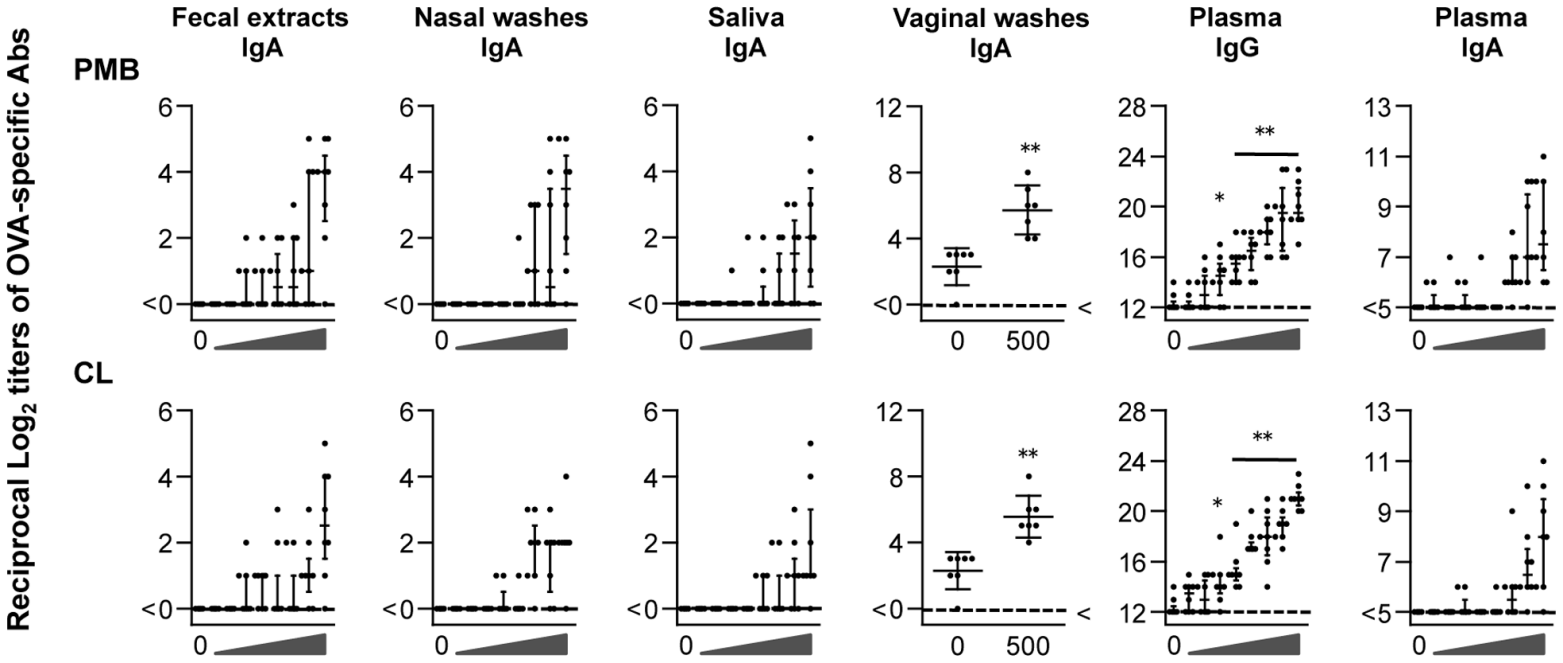
OVA-specific Ab responses in mucosal secretions and plasma. Mice were intranasally immunized with ovalbumin (OVA) plus 0, 2.5, 5, 10, 25, 50, 100, 250, or 500 µg of PMB or CL 3 times at weekly intervals. Mucosal secretions and plasma were collected at 1 week after the last immunization, and the OVA-specific IgA and IgG Ab levels were determined by endpoint ELISA. Each dot represents an individual mouse. Horizontal and vertical bars indicate mean ± standard deviation (SD) and median ± interquartile range (IQR), respectively, from 2 independent experiments (with 8 mice in each experimental group). Significant differences between the OVA alone and OVA plus PMB or CL groups are indicated with asterisks (*p<0.05 and **p<0.005).

While the titers of OVA-specific IgA Abs in the fecal extracts, nasal washes, and saliva from mice that were intranasally immunized with OVA alone were below the detection limit, the titers were increased by dose escalation of polymyxins (Fig. 2). Significantly, the titers of OVA-specific IgA Abs in vaginal washes from mice immunized with OVA plus 500 µg of either PMB or CL were higher, compared to mice immunized with OVA alone. We also observed that the levels of OVA-specific IgG and IgA Abs in plasma were increased by administration of polymyxins in a dose-dependent manner. In addition, there were no significant differences in the titers between PMB- and CL-immunized groups.

**Figure 3:**
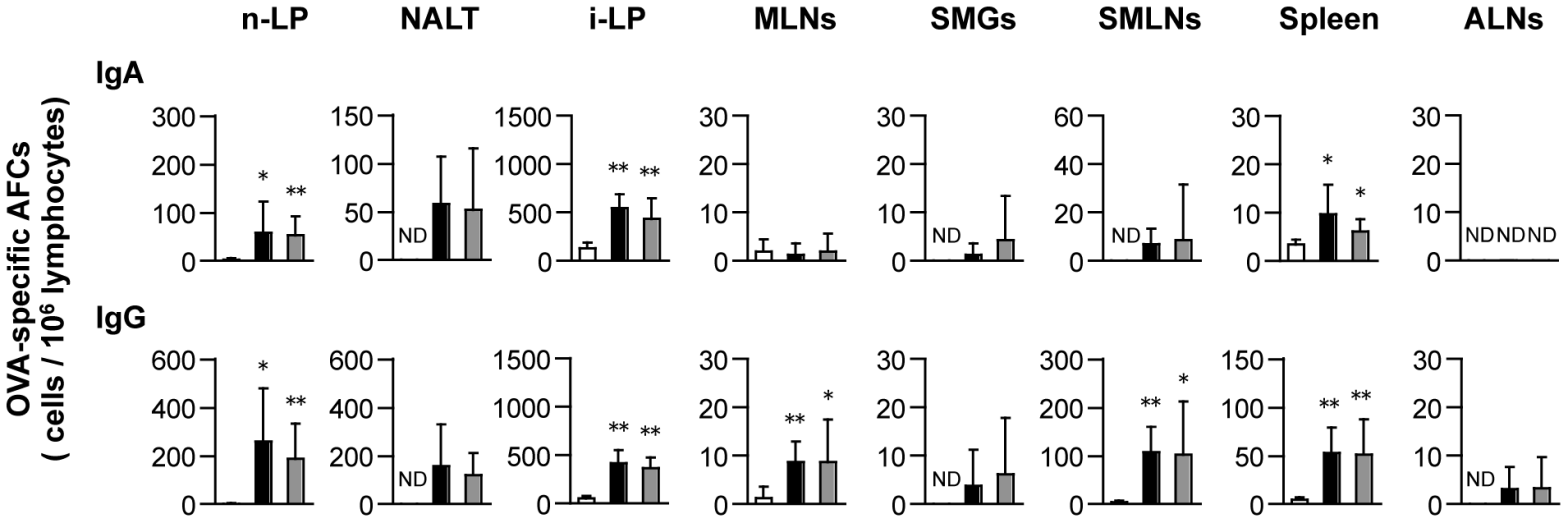
Analysis of OVA-specific AFCs in the mucosal and systemic tissues. Mice were intranasally immunized with OVA either alone (open column) or with 500 µg of PMB (closed) or CL (gray). One week after the last immunization, mononuclear cells isolated from various mucosal and systemic tissues were analyzed using an OVA-specific ELISPOT assay. n-LP, nasal lamina propria; NALT, nasopharynx-associated lymphoid tissue; i-LP, small intestinal lamina propria; MLNs, mesenteric lymph nodes; SMGs, submandibular glands; SMLNs, submandibular lymph nodes; and ALNs, axially lymph nodes. Data represent mean ± SD values of the number of AFCs/10^6^ cells from 3 independent experiments (with 12 mice in each experimental group). Significant differences between the OVA-alone and OVA plus PMB or CL groups are indicated with asterisks (* *p*<0.05 and ** *p*<0.005). ND, not detected.

To further confirm OVA-specific Ab responses at the cellular level, we compared the number of OVA-specific AFCs induced in mucosal (n-LP, NALT, i-LP, MLNs, SMGs, and SMLNs) and systemic (spleen and ALNs) lymphoid tissues. OVA-specific AFCs were detected in all mucosal tissues tested and spleen from mice immunized with OVA plus PMB or CL (Fig. 3). The number of OVA-specific IgA and IgG AFCs in the n-LP, i-LP, and spleen induced by intranasal immunization with OVA plus PMB or CL were significantly higher than those in mice immunized with OVA alone. These findings suggest that intranasally administered PMB and CL can act as a mucosal adjuvant to induce OVA-specific humoral immune responses in both mucosal and systemic tissues.

### Establishment of an ELISA System for Quantification of Anti-PMB Abs in Plasma

**Figure 4:**
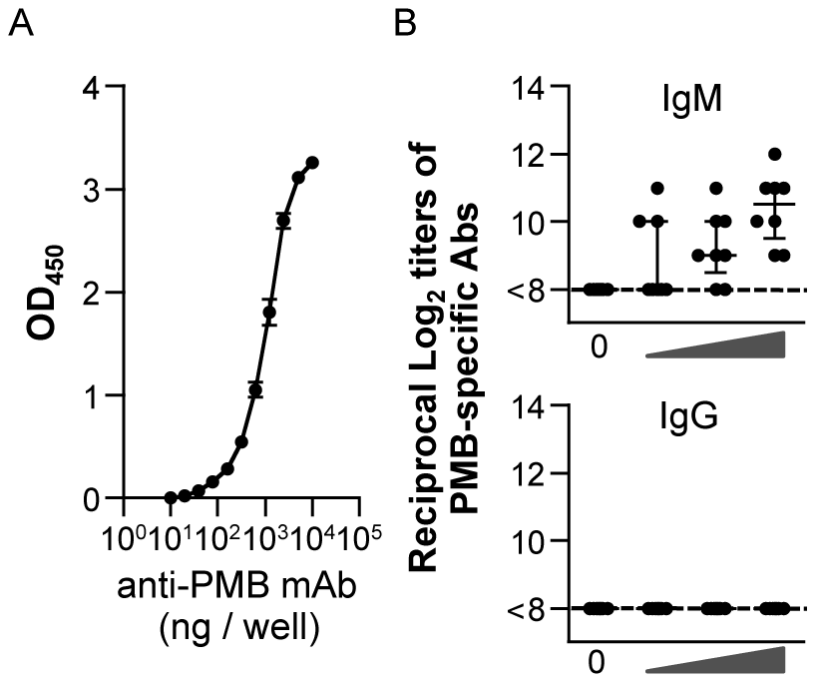
PMB-specific Ab responses in plasma. *A.* The PMB-specific Ab titers were determined by ELISA. To establish the ELISA system, 1,000 ng/well of anti-PMB mAb and twofold serial dilutions were tested. Standard anti-PMB was titrated on PMB-coated plates with a sensitivity of 20 ng/well. *B.* Plasma samples from mice intranasally immunized with OVA plus 0, 5, 50, or 500 µg of PMB 3 times at weekly intervals were assessed by PMB-specific endpoint ELISA. Each dot represents an individual mouse. Horizontal and vertical bars indicate median and IQR, respectively, from 2 independent experiments (with 8 mice in each experimental group).

Since Abs to PMB were produced in rodents immunized with PMB coupled to a carrier [39], [40], we assessed whether anti-PMB Abs were induced in mice immunized intranasally. We first established an ELISA system to detect anti-PMB Abs. Titration of anti-PMB Abs gave a sigmoid curve with a measurement interval between 10^1^ and 10^4^ ng/well (Fig. 4A). One week after the last immunization, the levels of PMB-specific IgM and IgG Abs in plasma were examined by this ELISA system. PMB-specific IgM Abs were detected in plasma from mice immunized with OVA plus PMB, whereas the titers of PMB-specific IgG Abs in plasma from all immunized mice were below the detection limit (Fig. 4B).

Since anti-PMB Abs were detected in immunized mice, we examined whether preexisting anti-PMB immunity would reduce the adjuvant function. We intranasally administered 500 µg of PMB thrice at weekly intervals and thereafter 100 µg OVA plus 500 µg of PMB. At 1 week after the last immunization of OVA plus PMB, the titers of OVA-specific Abs in plasma and mucosal secretions were compared, and no differences were observed between preadministered and naïve groups (data not shown).

### Assessment of Long-Lasting OVA-Specific Ab Production by Intranasal Immunization

**Figure 5:**
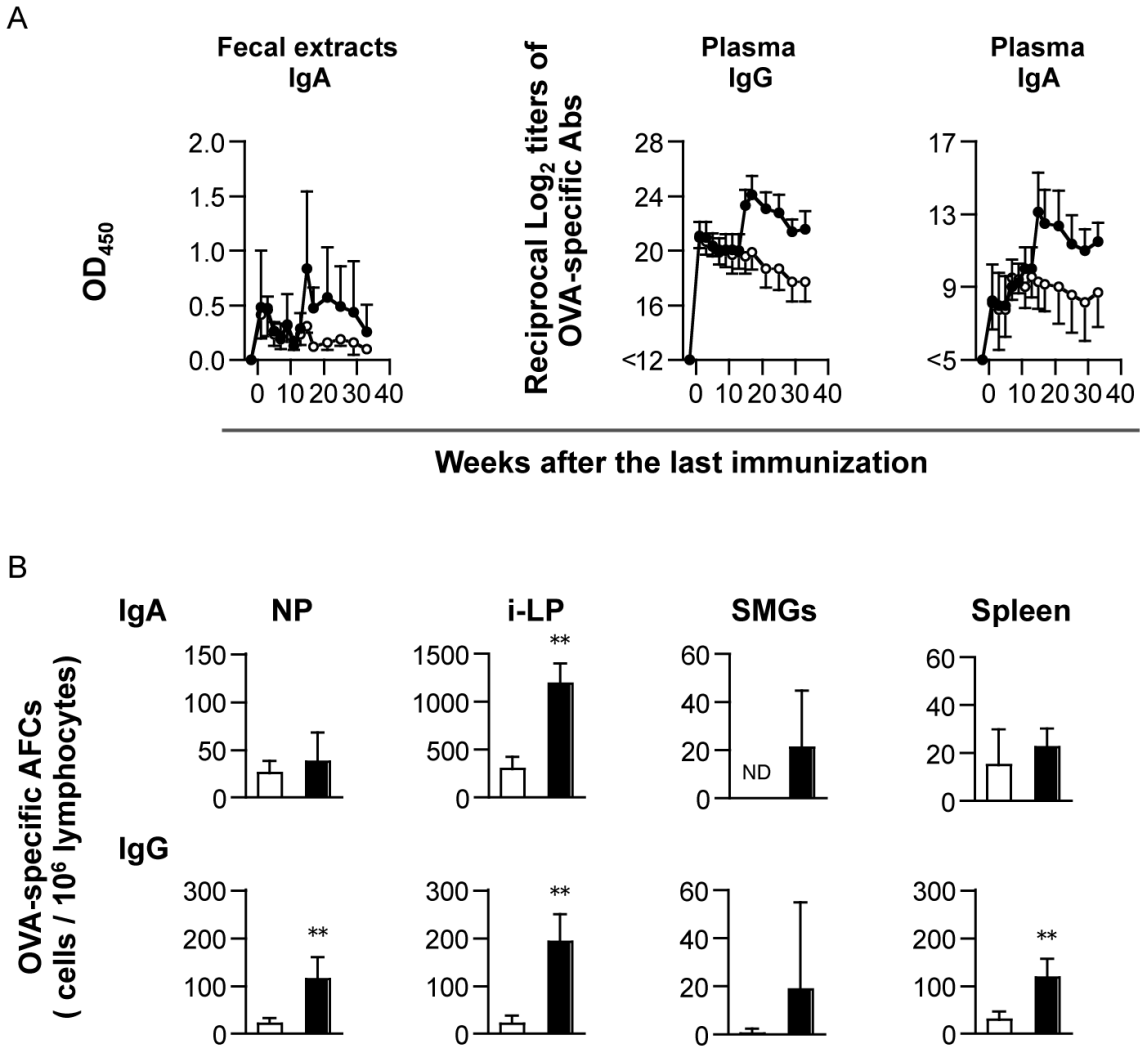
Long-lasting humoral immune responses by intranasal immunization of OVA plus PMB. Mice were intranasally immunized with OVA plus 500 µg of PMB 3 times at weekly intervals. One group was intranasally boosted with OVA plus 500 µg of PMB at 13 weeks after the last immunization (closed circle), whereas the other group was intranasally administered with normal saline (open circle). *A.* The levels of OVA-specific IgA Abs in fecal extracts, as well as IgG and IgA Abs in plasma, were determined by ELISA. Data represent mean ± SD values from 8 mice in each experimental group. *B.* After 31 weeks, mononuclear cells isolated from the n-LP, i-LP, SMGs, and spleen were determined using an OVA-specific ELISPOT assay. Data represent mean ± SD values of the number of AFCs/10^6^ cells from 8 mice per experimental group. Significant differences between non-boosted (open column) and boosted (closed column) groups are indicated with asterisks (* *p*<0.05 and ** *p*<0.005). ND, not detected.

Induction of immune memory is an important factor in strategic approaches to the development of a new vaccine adjuvant. We investigated the duration of OVA-specific long-lasting humoral immune responses in mice that were intranasally immunized with OVA plus PMB. Increases in OVA-specific IgA Abs levels were observed in fecal extracts until 3 weeks after the primary immunization; after peaking, however, the levels were barely detectable without boost (Fig. 5A). Similarly, the levels of OVA-specific IgG Abs, though detectable in plasma at 1 week after the last immunization, continued to decline after peaking. In contrast, when a single intranasal booster of OVA plus PMB was given at 13 weeks, not only did the declining OVA-specific Ab levels in both fecal extracts and plasma bounce back to high titers within 1 week, but the high levels were also maintained for 31 weeks of testing. Moreover, OVA-specific IgA and IgG AFCs were detected in all tested tissues from boosted mice, even though the levels in SMGs were scarcely detectable without booster (Fig. 5B). Markedly, the numbers of OVA-specific IgA and IgG AFCs in i-LP were significantly increased by booster immunization.

### Total IgE Ab Responses

One drawback of using adjuvants such as CT is the induction of high IgE Ab responses to bystander Ags. A previous study reported that the level of total IgE Abs was 1,165 ng/mL in plasma of mice intranasally immunized with OVA plus CT [30]. In this current study, we observed that the concentrations of IgE Abs in plasma of mice intranasally immunized OVA plus 2.5–500 µg of PMB or CL were roughly equivalent to those in plasma of mice intranasally immunized OVA alone (68.3 ng/mL) (data not shown).

### Subacute Toxicity of Polymyxins by Intranasal Immunization

We must consider the possibility that intranasal vaccines could cause damage to the central nervous system (CNS). Some intranasal adjuvants have been developed to avoid the risks in mucosal tissues and CNS [29], [30]. Our previous study showed that in the nasal cavity of mice intranasally immunized with OVA and 0.5 µg of CT, advanced inflammatory responses (grade 3 or 4, with inflammatory layers ≥10 cells thick) were observed [30]. In this study, only grade 1 inflammatory responses (inflammatory layers<5 cells thick) were observed in the nasal cavity of all mice tested (data not shown). Moreover, the histological abnormality was not found in olfactory bulbs. Renal insufficiency represents a major adverse effect of the use of polymyxins by intramuscular [41], [42] or intravenous [43] administration. Histological findings of polymyxin-induced renal damage usually involve focal irregular dilatation of tubules, epithelial and polymorphonuclear cell cast formation, as well as degeneration and regeneration of epithelial cells. In addition, separation of tubules by loose collagenous tissue, suggestive of edema, has also been reported [44], [45]. However, in this study we did not observe these adverse manifestations in kidney of mice intranasally administered with polymyxins (data not shown).

### Comparison of Immune Responses between C3H/HeN and C3H/HeJ Mice Immunized with OVA and PMB

**Figure 6:**
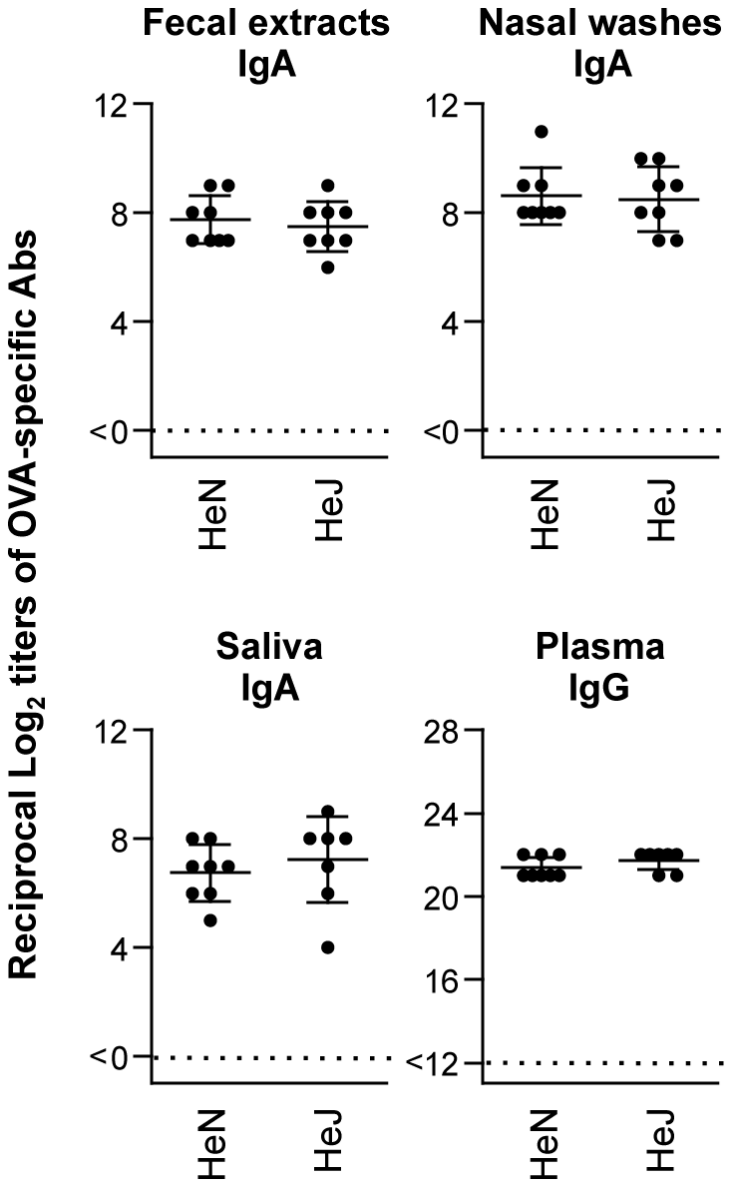
Comparison of immune responses between C3H/HeN and C3H/HeJ mice immunized with OVA and PMB. C3H/HeN and C3H/Hen mice were intranasally immunized with OVA plus 500 µg of PMB 3 times at weekly intervals. Mucosal secretions (fecal extracts, nasal washes, and saliva) and plasma were collected at 1 week after the last immunization. The OVA-specific IgA and IgG Ab levels were determined by OVA-specific endpoint ELISA. Each dot represents an individual mouse. Horizontal and vertical bars indicate mean and SD, respectively, from 2 independent experiments (with 8 mice in each experimental group).

While antibiotics induce cell death and lysis of gram-negative bacteria, lipopolysaccharide (LPS) might be set free from the surface of bacteria. LPS is a highly active immunomodulator and also a potent immunological adjuvant [46]. To determine whether LPS affects polymyxin adjuvanticity, we employed C3H/HeN and C3H/HeJ mice that carry wild-type and mutated toll-like receptor (TLR)-4, respectively [47], [48]. No significant differences in the levels of OVA-specific Abs in mucosal secretions and plasma were observed between C3H/HeN and C3H/HeJ mice intranasally immunized with OVA plus PMB (Fig. 6). These data show that the adjuvant potency of PMB in LPS-hyporesponsive C3H/HeJ mice was equivalent to that in normal LPS-susceptible C3H/HeN mice.

### Comparison of Adjuvanticity between Polymyxins and Their Analogues

**Figure 7:**
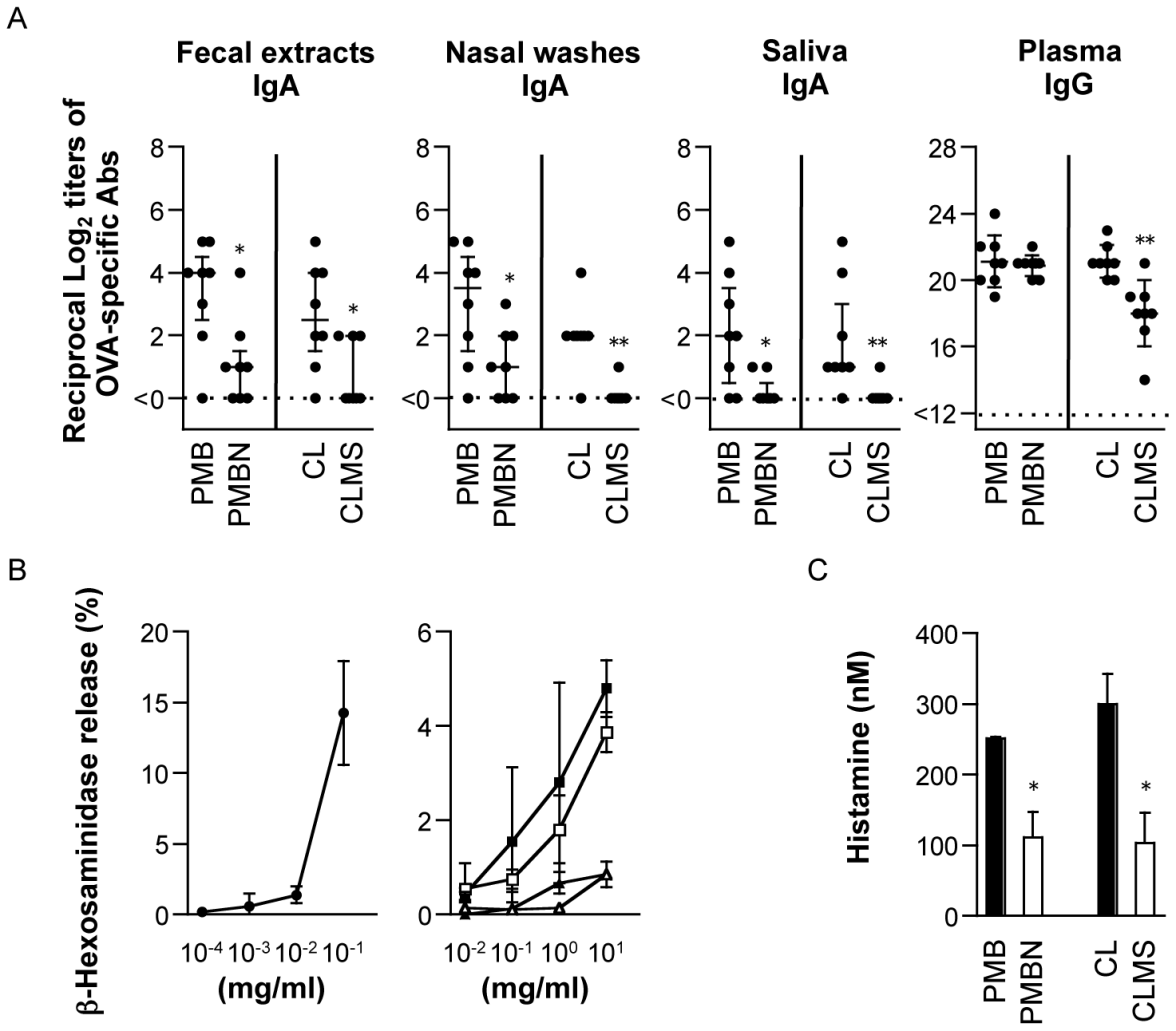
Comparison of immune responses and mast cell degranulation induced by polymyxins and their analogues. *A.* Mice were intranasally immunized with OVA plus 500 µg of PMB, PMBN, CL, or CLMS 3 times at weekly intervals. Mucosal secretions and plasma were collected at 1 week after the last immunization, and the OVA-specific IgA and IgG Ab levels were determined by OVA-specific endpoint ELISA. Each dot represents an individual mouse. Horizontal and vertical bars indicate either mean and SD or median and IQR, respectively, from 2 independent experiments (with 8 mice in each experimental group). Significant differences between the PMB and PMBN groups or between the CL and CLMS groups are indicated with asterisks (*p<0.05 and **p<0.005). *B.* Degranulation of MC/9 cells was assessed by a β-hexosaminidase release assay. Left panel, the percentages of β-hexosaminidase release elicited by compound 48/80 (a positive control); right panel, the percentages of β-hexosaminidase release stimulated by PMB (closed square), CL (open square), PMBN (closed triangle), or CLMS (open triangle). Data represent mean ± SD values from 3 different experiments. *C.* Histamine release of MC/9 cells was assessed by EIA. MC/9 cells were cultured with PMB, CL, PMBN, or CLMS. Data represent mean ± SD values from 3 different experiments. Significant differences between the PMB and PMBN groups or between the CL and CLMS groups are indicated with an asterisk (*p<0.05).

We evaluated the active center of polymyxin molecules for adjuvanticity by using their analogues: PMBN (generated by proteolytic cleavage using ficin or papain to remove the terminal amino acyl group [49]) and CLMS (produced by treating the 5 primary amine groups of α,γ-diaminobutyric acid residues in CL with formaldehyde and then sodium bisulfite [50]). As shown in Fig. 7A, the titers of OVA-specific IgA Abs in mucosal secretions from mice intranasally immunized with OVA plus PMB were significantly higher than those from PMBN-immunized mice, whereas the titers of plasma OVA-specific IgG Abs in both groups were roughly equivalent. In contrast, the titers of OVA-specific Abs in all tested samples from mice given OVA and CL were significantly higher than those in CLMS-immunized mice (Fig. 7A).

Nevertheless, the Ab titers in mucosal secretions induced by polymyxin analogues were increased, compared to the negative control. Specifically, the titers of OVA-specific IgG Abs in plasma of mice immunized with OVA plus PMBN or CLMS were significantly higher than those in mice immunized with OVA alone (*p*<0.005) (Fig. 7A vs. negative controls in Fig. 2).

To identify the differences between polymyxins and their analogues in the ability to enhance Ag-specific humoral immune responses, we assessed β-hexosaminidase activity and histamine release from MC/9 cells. As a positive control, the percentage of β-hexosaminidase release elicited by 0.1 mg/mL of compound 48/80 was approximately 14.2% (Fig. 7B, left panel). The percentages of β-hexosaminidase release stimulated by PMB or CL were increased in a dose-dependent manner, whereas those caused by PMBN or CLMS were less than 1% (Fig. 7B, right panel). Moreover, the released histamine levels in supernatants of MC/9 cells stimulated by PMB or CL were significantly higher than those stimulated by PMBN or CLMS (Fig. 7C), whereas the mean concentration of histamine in culture supernatants without stimulations was 1.1 nM (data not shown).

## Discussion

This current study provides direct evidence that intranasal coadministration of polymyxins with protein Ag enhances specific humoral immunity in mucosal and systemic compartments and also induces immunological memory in mouse models. While we propose that enhanced and long-lasting humoral immunity might be induced by polymyxins via activation of mast cells, the underlying mechanism has yet to be fully elucidated. In this study, we focused on the structure-based mechanism of polymyxin adjuvants. Since the general structure of polymyxins consists 2 parts, including a polar head and a fatty tail, we assessed which segment is important for the adjuvanticity by using polymyxin analogues. PMBN is a cationic peptide without a fatty tail, whereas CLMS is an amphiphilic surfactant with an anionic head. Both PMBN and CLMS were found to upregulate Ag-specific immunity and histamine release, indicating that each property of analogues might contribute to their adjuvanticity and that both cationic peptides and surfactants could become immune stimulators and candidate adjuvants [14], [15], [51]. Our results also show that the adjuvanticity of PMB and CL and their effects on mast cell degranulation were higher than those of PMBN and CLMS, suggesting that the potent adjuvanticity of PMB and CL might result from additive or multiplier effects of both cationic and amphiphilic structures.

Polymyxins are classified biosurfactants, which have several advantages over chemical surfactants, such as lower toxicity, higher biodegradability, and effectiveness at extreme temperatures or pH values [52], [53]. While more than 50 bioactive cyclic lipopeptides have been discovered in soil-associated bacteria, peptides with plural basic amino acids are rarely found [54]. Cyclic lipopeptides with diaminobutyric acid include only syringomycin and syringopeptin, besides polymyxins [54]. Interestingly, a correlation has been reported between the histamine-releasing activity of PMB analogues and the number of free positive charges on the molecules [27]. Since the structures of PMB and CL with 5 diaminobutyric acid residues are quite unique, they may be suitable for use as mucosal adjuvants.

Studies of vaccine development have pointed out the importance of assessing endotoxin contamination in the preparation of Ag and adjuvants [55], [56]. For instance, TLR activators usually elicit an inflammatory response, and there appears to be a direct relationship between the degree of inflammation induced and the adjuvanticity of many microbial products, such as LPS [57], [58]. Therefore, a critical issue that needs to be addressed is whether LPS, released from gram-negative bacteria by polymyxins as bactericidal antibiotics, could exert effects on TLRs supposedly present in mouse NALT. Our findings suggest that enhanced immune responses are not related to LPS liberated by polymyxins from normal bacterial flora in the nasal cavity and that polymyxins themselves possess the adjuvanticity.

Antibodies formed against protein adjuvants such as CT can bind themselves and potentially prevent their adjuvant effects [59]. Given the results of the production of anti-PMB IgM Abs by intranasal immunization with OVA plus PMB, a concern was raised as to the suitability of polymyxins for the general use as a vaccine adjuvant. However, our analysis of anti-OVA Ab responses in plasma and mucosal secretions indicates that preexisting anti-PMB immunity did not influence the adjuvanticity of polymyxins in a mouse model. Since there have been no studies examining anti-PMB Abs in patients using polymyxins, it will be necessary to confirm the production of anti-polymyxin Abs in clinical studies.

Polymyxins were used in intravenous therapy, but during 1950s and 1960s, concerns arose concerning adverse effects (nephrotoxicity, ototoxicity, and neuromuscular blockade) associated with their use [41]–[43]. However, polymyxins were brought back to clinical use because of the gradual increase in the infection incidence worldwide, due to the emergence of MDR gram-negative pathogens in hospitalized patients with considerable morbidity and mortality, as well as the unavailability of newer antimicrobial agents to combat these infections [22], [23]. Meanwhile, their administration by inhalation has been proposed as a possible useful alternative for treating lung infections [60]. Different modes have been used for the administration of polymyxins into the respiratory tract; among them, nebulization of the drug via a variety of different nebulizers, inhalation of dry powder, and endotracheal instillation are the commonest [61]–[65].

Polymyxins are very low absorption from the mucosa [40]. Indeed, the LD_50_ value in mice intravenously administered PMB was 5.4 mg/kg, whereas the LD_50_ value was 790 mg/kg when mice were orally administered PMB after fasting for 4 h [66]. Such a property of polymyxins might be advantageous for developing safer adjuvants administered via the mucosal route. Moreover, the maximum dosage of PMB in the current study was 500 µg/mouse. Since the body weight of mice used in our experiment was 16–18 g, the amount can be converted as 28–31 mg/kg, which is approximately one-25th of the LD_50_ value by oral administration. Although there have been no studies on the LD_50_ value in intranasally administered mice, we consider that polymyxins might be safe as a mucosal adjuvant. While clinical trials are required to investigate whether the polymyxin adjuvants are effective in humans, we present here that polymyxins are safe mucosal adjuvants.

Mucosal infectious pathogens kill children mostly under the age of 5 years and are responsible for some 10 million deaths of children annually. Because polymyxins have been clinically used in children and neonatal patients [67], [68], we propose that the use of polymyxins as mucosal adjuvants may provide important insights into the development of safer vaccines, especially for infants and children.
